# Endotracheal intubation in rabbits using a video laryngoscope with a modified blade

**DOI:** 10.1186/s42826-022-00130-7

**Published:** 2022-07-27

**Authors:** Yujin Kim, Hee Yeon Jeon, Insook Yang, Ji Hyun Kim, Jae Hee Chung, Ji-Hyeon Ju, Gyeonghun Kim, Jun Won Park, Seung Yeon Oh, Je Kyung Seong, Seung Hyun Oh, Young-Shin Joo

**Affiliations:** 1 Laboratory Animal Research Center, Institute of Biomedical Industry, The Catholic University of Korea, Seoul, Republic of Korea; 2 Department of Laboratory Animal, College of Medicine, The Catholic University of Korea, Seoul, Republic of Korea; 3 Department of Laboratory Animal Resources, Yonsei Biomedical Research Institute, Yonsei University College of Medicine, Seoul, Republic of Korea; 4 Department of Surgery, College of Medicine, Eunpyeong St. Mary’s Hospital, The Catholic University of Korea, Seoul, Republic of Korea; 5 Department of Surgery, College of Medicine, Seoul St. Mary’s Hospital, The Catholic University of Korea, Seoul, Republic of Korea; 6 Division of Rheumatology, Department of Internal Medicine, College of Medicine, Seoul St. Mary’s Hospital, The Catholic University of Korea, Seoul, Republic of Korea; 7 Laboratory of Developmental Biology and Genomics, BK21 Program for Veterinary Science, College of Veterinary Medicine, Seoul National University, Seoul, Republic of Korea; 8 Division of Biomedical Convergence, College of Biomedical Science, Kangwon National University, Chuncheon, Republic of Korea; 9 College of Pharmacy, Gachon University, Incheon, Republic of Korea

**Keywords:** Rabbits, Inhalation anesthesia, Endotracheal intubation, Video laryngoscopes, Modified blade, Modified stylet

## Abstract

Rabbits are being increasingly used as companion animals, and in research; thus, the need for proper veterinary care for rabbits has increased. Surgical access is more challenging in rabbits under inhalation anesthesia compared to other animals, such as dogs and cats. Rabbits have a very narrow and deep oral cavity, large incisors, and a large tongue. Moreover, their temporomandibular joint has limited mobility, making it more difficult to approach the larynx. Various methods have been proposed to overcome this difficulty. The video laryngoscope was introduced in 1999 and is useful when airway intubation is unsuccessful using a conventional laryngoscope. We postulated that a video laryngoscope with a modified size 1 Macintosh blade (McGrath MAC Video Laryngoscope, Medtronic, USA) would facilitate the intubation of New Zealand White rabbits. Sixteen specific-pathogen-free male New Zealand White rabbits weighing 3.45–4.70 kg were studied. All rabbits were intubated using the video laryngoscope. Typically, a 3.0 mm endotracheal tube was used for rabbits weighing < 4 kg, while a 3.5 mm tube was used in those weighing > 4 kg. During surgery, anesthesia was well maintained, and there were no major abnormalities in the animals’ conditions. No rabbit developed breathing difficulties or anorexia after recovering from anesthesia. We established an intubation method using a video laryngoscope with a modified blade and stylet in the supine (ventrodorsal) position and successfully applied it in 16 rabbits. It is useful for training novices and for treating rabbits in veterinary hospitals with few staff members and animal research facilities where there are insufficient human resources.

**Supplementary Information:**

The online version contains supplementary material available at 10.1186/s42826-022-00130-7.

## Background

Rabbits are popular companion animals worldwide, and are also important for research studies [1, 2]. The need for proper veterinary care for rabbits has increased in line with their growing popularity. However, surgery in rabbits is very risky compared to other animals, such as dogs and cats [3]. In particular, inducing inhalation anesthesia in rabbits is not easy because of the difficulties associated with endotracheal intubation. Rabbits have a very narrow and deep oral cavity, large incisors, and a large tongue. Moreover, their temporomandibular joint has limited mobility [4, 5].

Video laryngoscopes (VLs) have been widely used since their development in 1999 for patients in whom conventional laryngoscope (CL) intubation is difficult. Simple, easy-to-use VLs are now available at a comparatively low cost [6], and can be used to visualize the epiglottis in human medicine easily [7].

We postulated that a VL with a modified size 1 Macintosh blade (McGrath MAC VL, Medtronic, USA) would facilitate the intubation of New Zealand White rabbits.

## Main text

### Animals

Sixteen specific pathogen-free male New Zealand White rabbits weighing 3.45–4.70 kg were used (15 from Granja San Bernardo, Spain and one from Qingdao Kangda Rabbit Development, China). The animals were housed individually in stainless steel cages that could be auto-washed and auto-watered and maintained on a 12 h light–dark cycle (lights off at 8 pm). The room temperature was 18–26 °C, and humidity was 50 ± 10%. The food was Lab Rabbit Chow; antibiotic-free/gamma sterilized diet (38302AF, Purina®, Korea) and tap water were offered ad libitum. A mineral block (Bunny Blocks; Bio-Serv, USA), wood block (TAPVEI®, Estonia), hay, and fresh organic vegetables were provided for environmental enrichment.

The VL intubation technique was developed in six rabbits during a rabbit practical training workshop held by the Laboratory Animal Research Center (LARC), Institute of Biomedical Industry and Department of Laboratory Animal (DOLA), College of Medicine, The Catholic University of Korea. Then it was used to intubate 10 rabbits scheduled for cardiovascular, orthopedic, and gastrointestinal surgery (3 rabbits each) and plastic surgery (1 rabbit).

All surgical interventions and pre- and post-surgical animal care were conducted in accordance with the Animal Protection Act, Laboratory Animal Act, and Guide for the Care and Use of Laboratory Animals of the Institutional Animal Care and Use Committee (IACUC) of the College of Medicine, Catholic University of Korea Songeui Campus (approval CUMS-2020-0001-08).

The IACUC and Department of Laboratory Animals of at the Catholic University of Korea (Songeui Campus) were accredited as a Korea Excellent Laboratory Animal Facility (No. 10) by the Korea Ministry of Food and Drug Safety in 2017, and acquired full accreditation from the Association for Assessment and Accreditation of Laboratory Animal Care International in 2018.

### Video laryngoscope and intubation technique

**Fig. 1:**
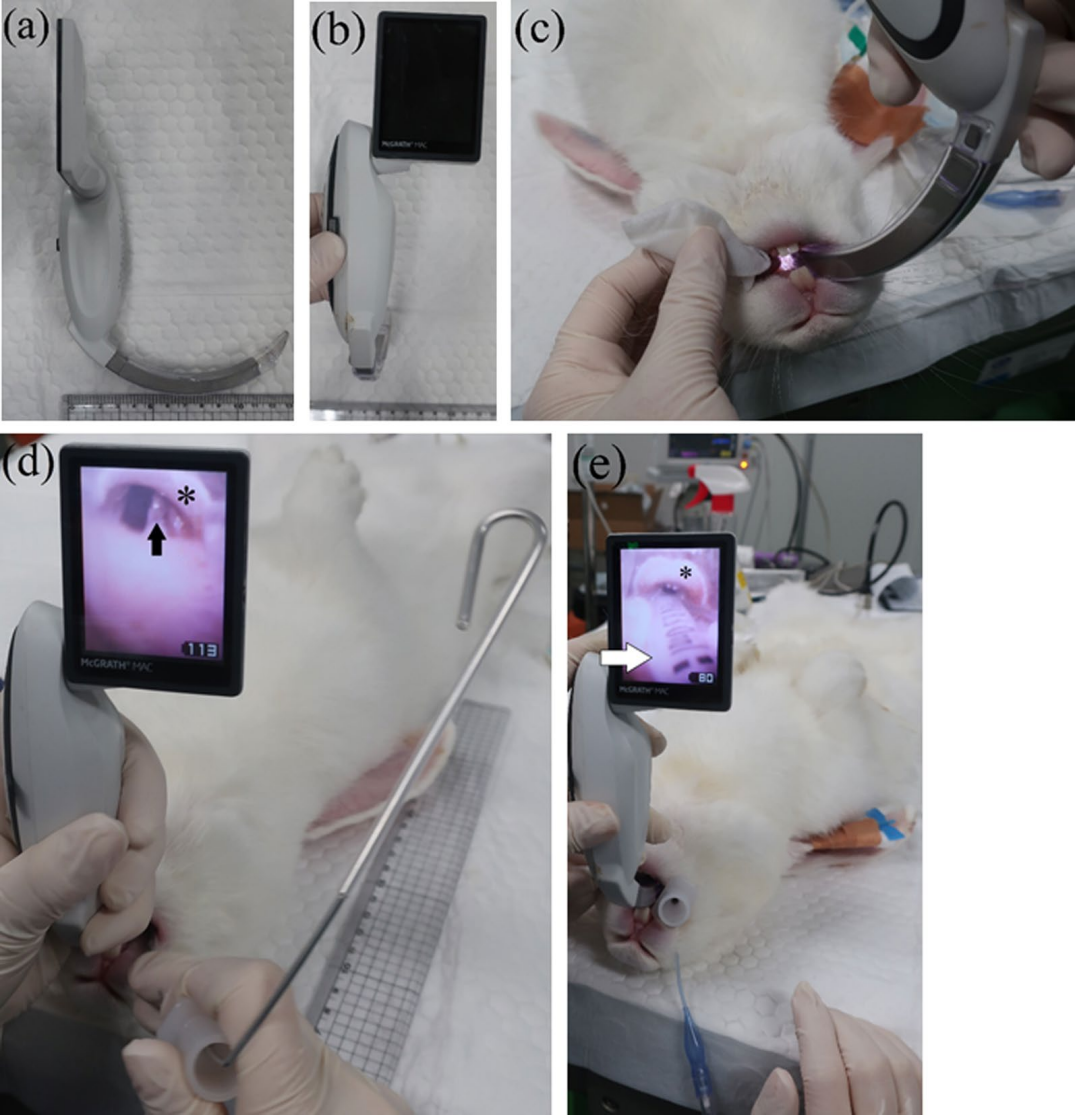
View of the video laryngoscope and modified blade and intubation with the video laryngoscope showing **a** side view of the laryngoscope with the blade attached, **b** the screen, **c** the tongue wrapped in moistened gauze and pulled toward the left side of the mouth with the left hand, and the laryngoscope blade inserted under the tongue through the right side of the mouth with the right hand. **d** The ET tube was inserted into the right side of the mouth. The epiglottis (*) and vocal folds (black arrow) were identified using the VL. The blade was inserted between the upper and lower incisors. **e** ET tube (white arrow) inserted through the vocal folds into the trachea

**Fig. 2:**
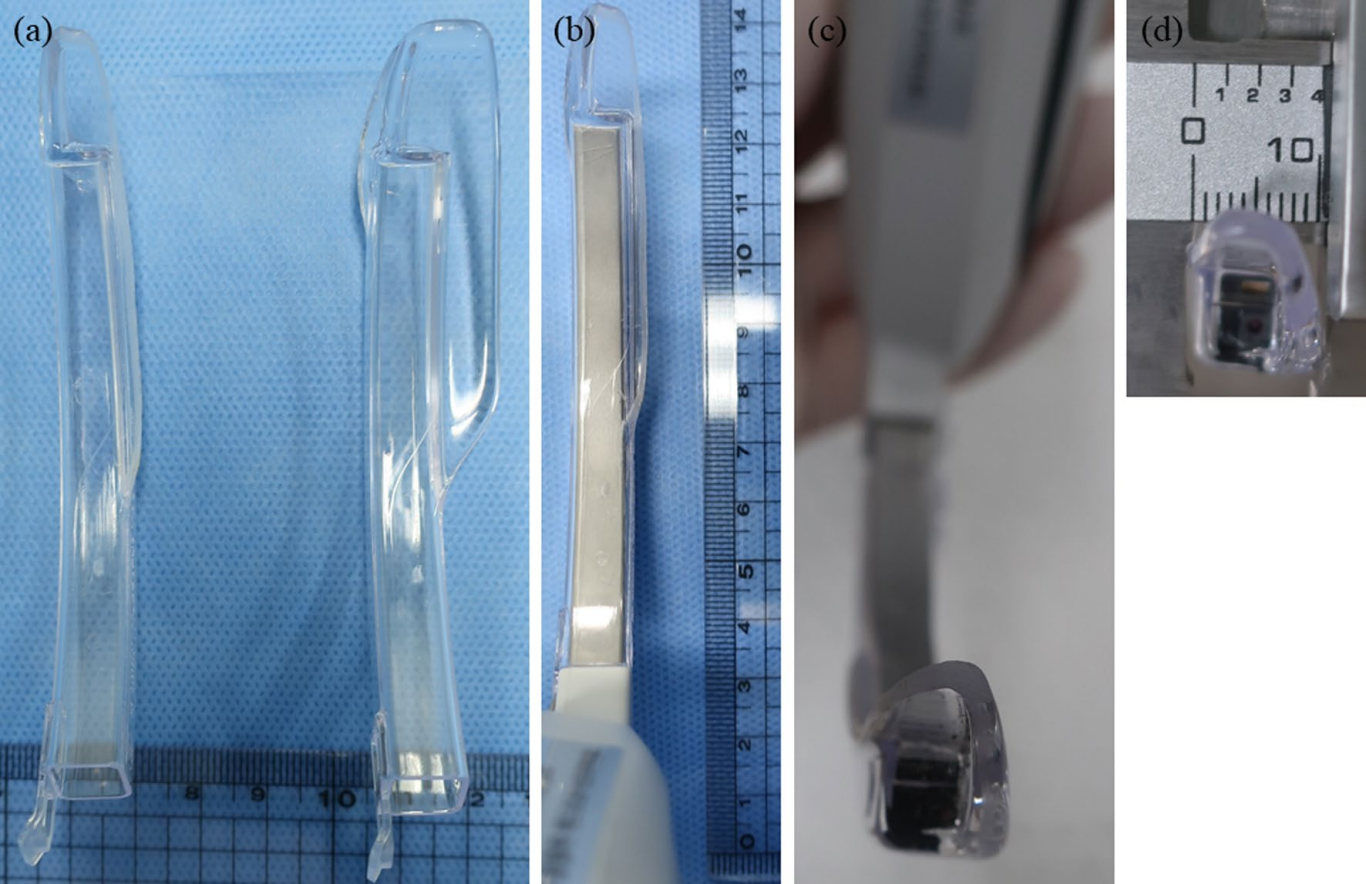
Laryngoscope blade. **a** Modified (left) and original blade (right); **b** modified video laryngoscope blade; **c** the blade in front of the lens and **d** size of modified blade: width 1 cm, length 1 cm). A McGrath MAC #1 blade (**a**) (right) is too wide to insert into a rabbit’s mouth, so the wing was cut off (for a blade size of ~ 1 cm) with a cutting machine (28,170 Mini bend saw; PROXXON, Japan) and the edge was smoothed with a grinder (Dremel 8220; Bosch Poser Tools, the Netherlands) (**b**). To secure the field of view, anti-fogging solution (Fig. 3f, SWAVE Anti-fog solution, 10 mL; GANA, Pusan, Korea) was applied to the blade in front of the lens (**c**), (**d**)

**Fig. 3:**
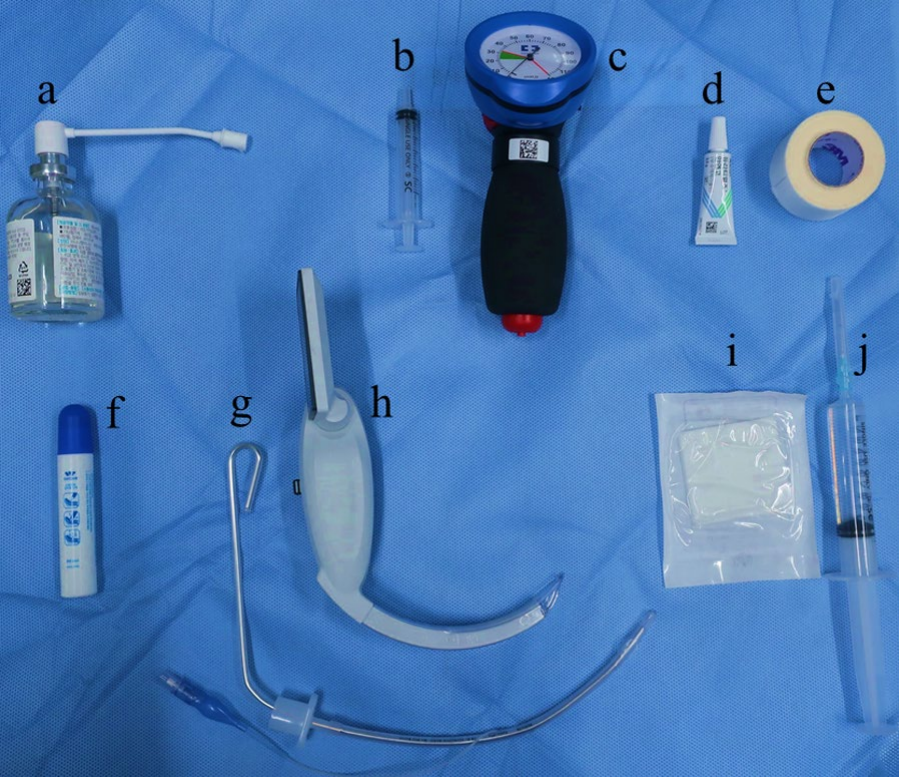
Equipment assembled before endotracheal intubation. **a** Lidocaine spray; **b** 3 mL syringe marked at the 20–30 cmH2O position; **c** manometer; **d** eye ointment; **e** cotton tape used for fixing the anesthesia circuit; **f**, anti-fogging solution of polymer surfactant components that satisfies the criteria for detecting harmful ingredients of Korea Conformity Laboratories; **g** 3.5 mm endotracheal tube with a 10Fr stylet; **h** video laryngoscope; **i** 5 × 5 cm gauze for wrapping the tongue, and **j** 10 mL syringe filled with normal saline to soak the gauze

Figure 3 shows the equipment used for rabbit intubation. A McGrath MAC VL (Fig. 1) was used with a Macintosh #1 blade that had been modified by cutting off the wing and smoothing the edge to form a blade measuring ca. 1 cm (Fig. 2). To secure the field of view, 10 mL SWAVE anti-fogging solution (GANA, Korea) was applied to the blade in front of the lens (Fig. 3f).

Shiley Hi-Contour Oral/Nasal endotracheal (ET) tubes (Covidien, USA) with inner diameters (IDs) of 3.0, 3.5, and 4.0 mm and respective outer diameters (ODs) of 4.3, 4.9, and 5.6 mm were used without being cut (REF 107-30, 107-35, and 107-40, respectively). A 10Fr stylet (Shiley™ Intubating Stylet, Covidien, USA) was inserted inside the 3.5 and 4.0 mm ID ET tubes and a 10Fr stylet with the rubber covering stripped from the distal 60% of the stylet using long-nose pliers was used for the 2.5- and 3.0 mm ID ET tubes. Then the stylet was bent approximately 90° at the junction with the ET tube connector and the part inside the ET was bent into a gentle curve (Fig. 3g).

Before use, the modified blade and stylet were sterilized using ethylene oxide gas (Steri-Vac; 3M, USA) or hydrogen peroxide (STERRAD NX sterilizer; Johnson & Johnson, USA).

**Fig. 4:**
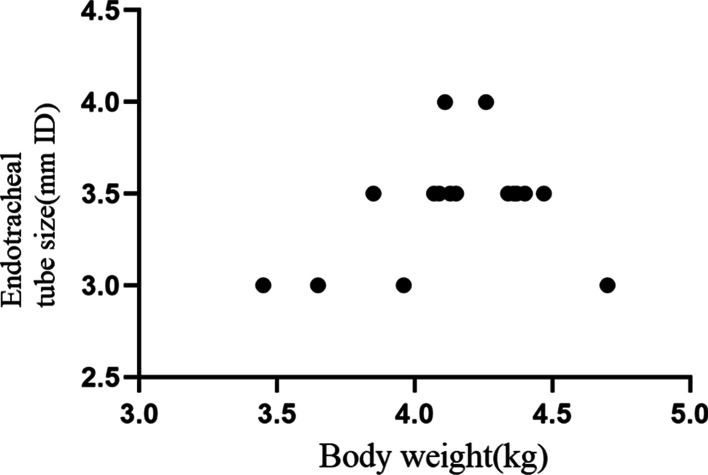
Endotracheal (ET) tube size and body weights of the 16 rabbits.Generally, a 3.0 mm ET tube was used for rabbits weighing < 4 kg, while a 3.5 mm was used in animals weighing > 4 kg. (ID, internal diameter)

The ET tube and stylet were selected according to bodyweight, but smaller sizes were used if it was difficult to pass the tube through the vocal fold during intubation (Fig. 4) [8]. The seal of the ET tube cuff was tested with a 3 mL syringe before use. Each rabbit was placed in a ventrodorsal position on the operating table. The trachea and head were maintained in a straight line during intubation.

Intubation was performed when the numerical rating scale score for induction and endotracheal intubation quality was 3 or 4 [1]. We wrapped the rabbit’s tongue in 5 × 5 cm gauze (Panamedic, Korea) moistened with normal saline (Dai Han Pharm., Korea) and pulled it toward the left side of the mouth. The VL was positioned under the tongue through the right side of the mouth (Fig. 1c). The blade was advanced while checking the internal structure of the oral cavity on the VL monitor. The blade was passed between the molars or under the tongue, and then placed between the upper and lower incisors. The blade was advanced slowly until the soft palate or epiglottis was visible. If the tip of the blade was too close to the epiglottis, there was no space for the ET tube. After grasping the VL with the left hand, lidocaine (Firson, Korea) was sprayed on the epiglottis and vocal folds. The ET tube and stylet were inserted into the right side of the rabbit’s mouth (Fig. 1d). At this point, the tongue was on the left side of the oral cavity, the VL blade was between the upper and lower incisors, and the spraying device or ET tube was inserted into the space between the right upper and lower teeth. The tip of the ET tube was in front of the epiglottis on the monitor, and the tube was gently pushed through the gap in the vocal folds. If resistance was felt, more anesthetic was sprayed onto the vocal folds, or the tube was turned slightly to move the tip toward the gap between the vocal folds (Additional file 1: Video 1). When the ET tube had passed easily between the vocal folds, the epiglottis and vocal folds were observed around the tube (Fig. 1e). After confirming intubation, the VL and stylet were removed gently. At this time, when the animal exhales, it can be seen in the ET tube (Additional file 1: Video 1). When checked at the lip, the distance from the patient end of the ET tube was around 15 cm. The cuff of the ET tube was inflated at 20–30 cmH2O with a 3 mL syringe or monometer (109-02; Covidien, Germany) [9]. The ET tube was connected to a Y-piece and the end-tidal partial pressure of carbon dioxide (PE´CO_2_) was detected on a monitor (Additional file 1: Video 2). The tube was fixed to the back of animal’s head using gauze. An inhalation anesthesia machine (AVANCE; GE Healthcare, USA) and patient monitor (BM3VET Pro; Bionet, Korea or AM; GE Healthcare, USA) were used to maintain anesthesia.

Researchers and laboratory animal veterinarians (LAVs) intubated six of the rabbits using a VL in a practical techniques workshop about handling, drug administration, blood collection, anesthesia and pre-, peri-, and postoperative care. Subsequently, three LAVs at LARC applied the method and equipment (Fig. 3) to 10 rabbits undergoing surgery. Figure 4 shows the size of the ET tube. Typically, a 3.0 mm ET tube was used for rabbits weighing < 4 kg, while a 3.5 mm was used in animals weighing > 4 kg (Fig. 4).

**Fig. 5:**
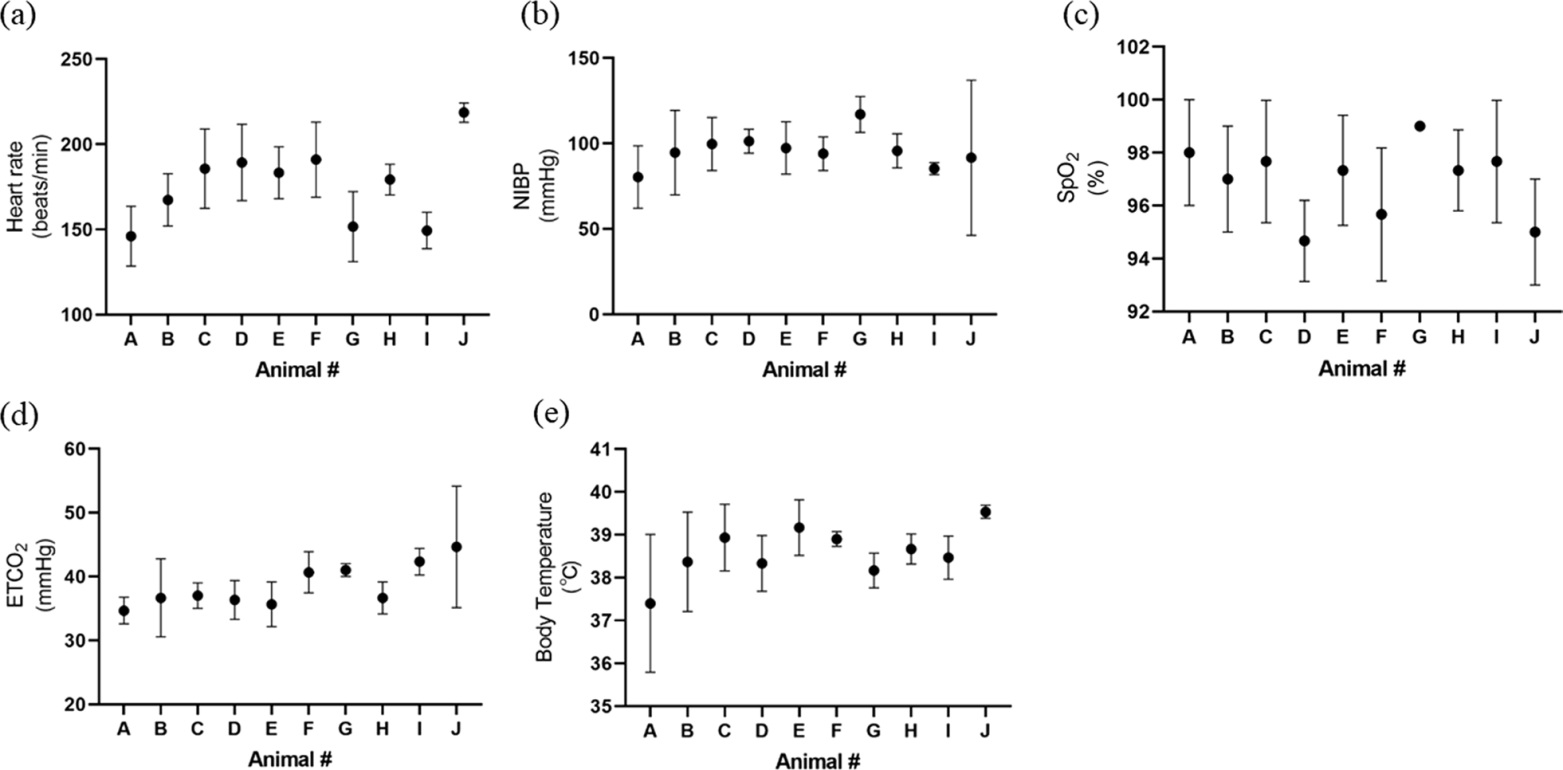
Mean data obtained during surgery on 10 rabbits. **a** Heart rate, 176.2 ± 22.8 beats per minute; **b** non-invasive blood pressure (NIBP), 95.7 ± 9.8 mmHg; **c** peripheral oxygen saturation (SpO2), 96.9 ± 1.4%; **d** end-tidal CO_2_ pressure (ETCO_2_), 38.6 ± 3.3 mmHg; **e** body temperature, 38.6 ± 0.6 °C

**Fig. 6:**
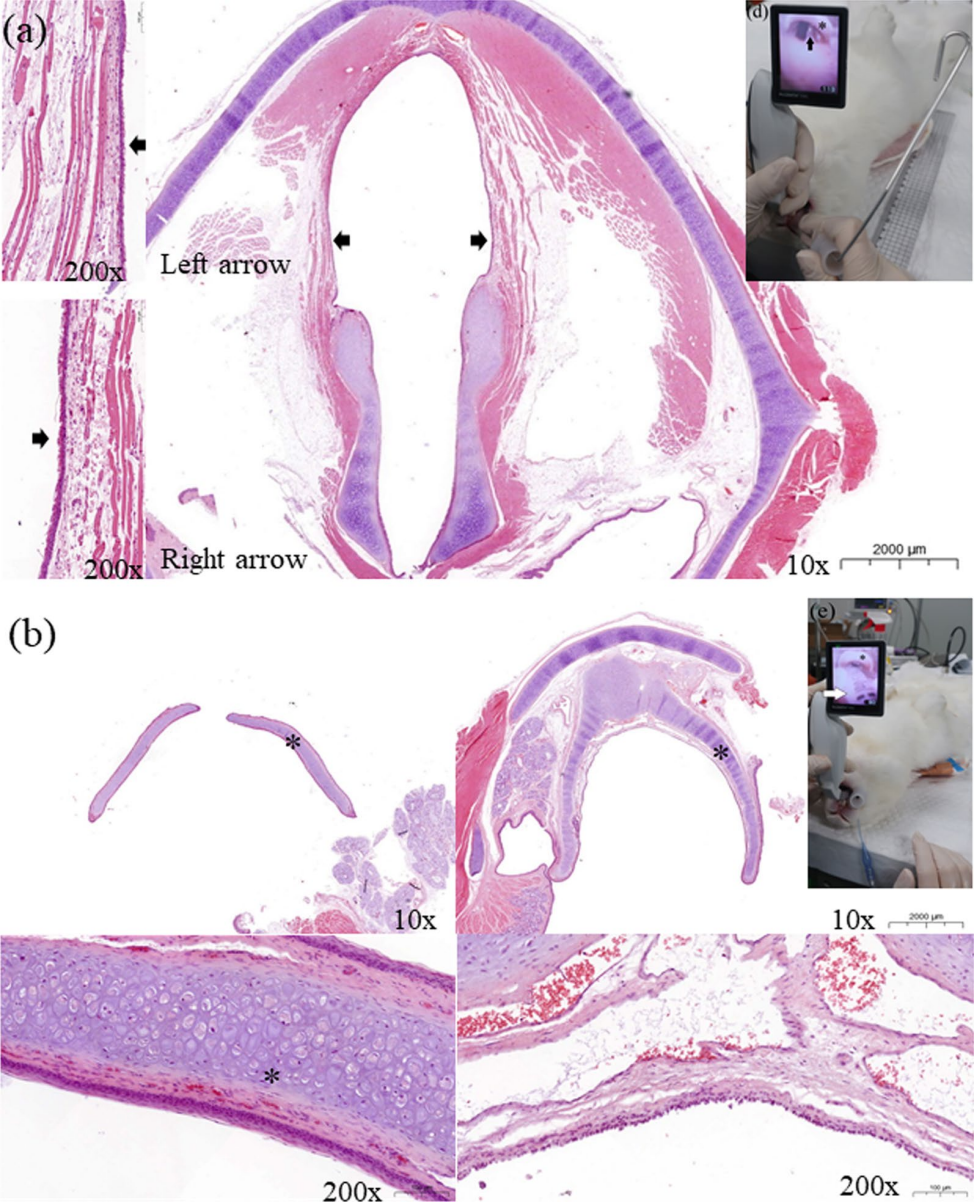
Oropharynx of a rabbit 2 weeks after intubation. HE staining of the **a** vocal folds (black arrow) and **b** epiglottis (*). No abnormalities were identified

**Fig. 7:**
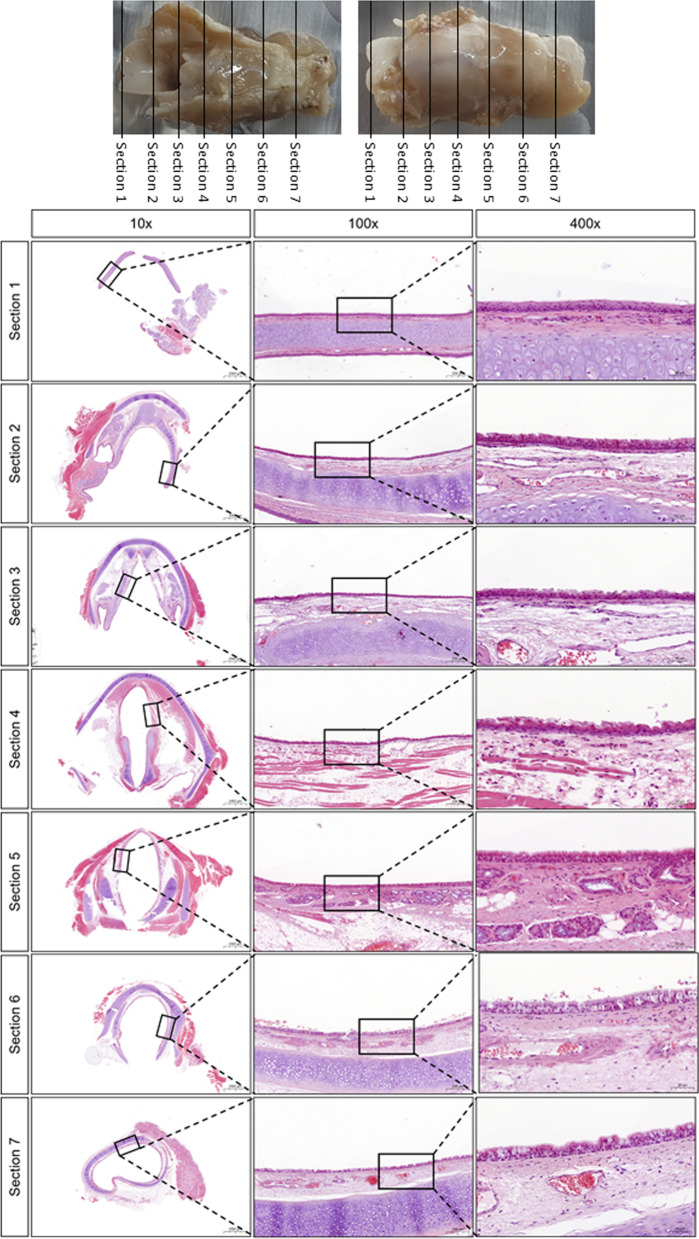
Oropharynx of a rabbit 2 weeks after intubation. HE staining of a transverse section of the oropharynx. No abnormalities were identified

The heart rate, blood pressure, SpO_2_, PE´CO_2_, and body temperature were recorded every 10–15 min during surgery (Fig. 5). During surgery, anesthesia was maintained well and no major abnormalities developed in the animals’ condition. No rabbit developed breathing difficulties or anorexia after recovering from anesthesia. All rabbits were euthanized after the experiment. No abnormalities were identified in the oropharynx of one of rabbit, which was sacrificed 2 weeks after intubation, at the end of the experiment (Figs. 6, 7).

### Anesthesia

Blood was analyzed before surgery. Local anesthetic cream (Recipharm Karlskoga, Sweden) was applied to the marginal ear vein of each rabbit 15–20 min before cannulation. Then glycopyrrolate (0.01 mg kg^−1^ subcutaneously [SC]; Myungmoon Pharm, Korea) was injected using a 1 mL syringe with a 26G needle. After 10–15 min, xylazine (5 mg kg^−1^ SC; Bayer Korea, Korea) was administered using a 1 mL syringe. Once the animal had been sedated, it was preoxygenated using a face mask, and the marginal ear vein was cannulated using a 24G intravenous (IV) catheter (BD, USA). Then a Luer-Lok adapter (Hyupsung Medical, Korea) was attached to the IV catheter and flushed with 0.9% normal saline (Dai Han Pharm., Korea). Next, ketoprofen (3 mg kg^–1^ IV; SDC Pharm., Korea) or tramadol (5 mg kg^–1^ IV; Yuhan, Korea) was injected as analgesia depending on the surgery. Cefazolin (33 mg kg^–1^ IV; Chong Kun Dang Pharmaceutical, Korea) was given as an antibiotic. Ketamine (10 mg kg^–1^ IV; Yuhan, Korea) or tiletamine and zolazepam (15 mg kg^–1^ intramuscularly [IM]; Virbac Laboratories, France) were administered to induce anesthesia. Inhalation isoflurane (1–3%; Hana Pharm, Korea) or sevoflurane (1–3.5%; Hana Pharm, Korea) was used as the inhalation anesthetic in 10 rabbits undergoing various surgeries. Eye ointment (Novartis, Belgium) was applied after inducing anesthesia to prevent the cornea from drying. Between drug injection (1–2 mL IV) and fluid therapy (5–10 mg kg^–1^ h^–1^ IV) during surgery, 0.9% normal saline was administered slowly [10–12].

### Monitoring

The PE´CO_2_, peripheral oxygen saturation (SpO_2_), body temperature, and noninvasive blood pressure (NIBP) were monitored during the surgery in 10 rabbits; an electrocardiogram (ECG) was also performed [11, 12].

### Statistical analysis

GraphPad Prism (ver. 9.3.1; GraphPad Software, USA) was used to perform the one-sample *t-*tests used for the statistical analysis in this study. A *p*-value < 0.033 was considered significant.

## Conclusions

Various complications have been reported during or after anesthesia for rabbit intubation, such as damage to or bleeding of the larynx, laryngeal spasm [1, 5]. Most of these complications are caused by multiple intubation attempts without a clear view when using a CL. These can be mitigated if intubation is carried out under direct view. Novice LAVs with less than 3 years of experience who could not intubate with a CL intubated using a VL without problems (Table 1). An expert with more than 15 years of experience took less time to find the epiglottis using a VL, but the intubation time was faster when using a CL (Table 1). VLs appear to be a good method for finding the epiglottis, and novices will be helped when intubating rabbits, although more subjects are needed to verify these results.

**Table 1:** Comparison of intubation using a CV and LV by an expert and novices

Type	Checkpoint	Expert (LAV, *n* = 1)	Novice (LAV, *n* = 2)
Conventional laryngoscope	Time to find the epiglottis	10 ± 1 s	141.8 ± 37.1 s
	Time for intubation	16.3 ± 1.2 s	Failed
	Intubation success rate	100%	0%
	Degree of damage	None	Edema and bleeding of the oropharynx
Video Laryngoscope	Time to find the epiglottis	5.3 ± 1.2 s	15.7 ± 4.8 s
	Time for intubation	26.3 ± 8.5 s	46.2 ± 18.8 s
	Intubation success rate	100%	100%
	Degree of damage	None	None

The expert (*n* = 1) is a LAV with more than 15 years of experience and the novices (*n* = 2) had less than 3 years of experience. The time required to find the epiglottis and perform intubation was the average for the three cases in which intubation was performed within 5 min after inducing anesthesia. The expert required less time to find the epiglottis when using a VL, but the time to intubate was faster for a CL. In the novice, the time needed to find the epiglottis was much shorter and intubation was successful when using a VL, but was not successful when using a CL. In both the expert and novices, intubation was possible without damaging the oropharynx when a VL was used, but swelling and bleeding were observed when the novices used a CL

Once mastered, a VL can be used for intubation without assistance because the intubation is performed in the supine rather than the sternal position. This is useful for treating rabbits in veterinary hospitals with few staff members or animal research facilities where there are insufficient human resources.

To adapt a VL for rabbit intubation, the blade was cut (28,170 Mini bend saw; PROXXON, Japan) to an appropriate size for the rabbit mouth under the guidance of a biomedical engineer (Fig. 2). The cut edge was smoothed with a grinder (Dremel 8220; Bosch Poser Tools, the Netherlands) to prevent mouth injuries. The stylet used for the 3.0 mm ET tube was also modified because a 6Fr stylet provided inadequate stability.

As the rabbits in this study all weighed > 3 kg, further studies of lighter animals are needed. After establishing this method in six rabbits in the LARC and DOLA workshop, intubation using video laryngoscopy was performed in 9 surgeries in 2020 and 15 surgeries in 2021.

It is hoped that the modified VL components (blade and stylet) described here will be commercialized. We also hope to produce additional modified products for veterinary medicine and research.

## Supplementary Information

**Additional file 1**: **Video 1** Intubation with the video laryngoscope.

**Additional file 2**: **Video 2** Intubation with the video laryngoscope.

## Data Availability

All relevant data are presented in the article. The datasets used and/or analyzed during the current study are available from the corresponding author on reasonable request.

## Abbreviations

AAALAC: Association for assessment and accreditation of laboratory animal care
CL: Conventional laryngoscope
DOLA: Department of laboratory animals
IACUC: Institutional animal care and use committee
ID: Inner diameter
IM: Intramuscularly
IV: Intravenously
ECG: Electrocardiogram
ET: Endotracheal
LARC: Laboratory animal research center
LAV: Laboratory animal veterinarian
NIBP: Noninvasive blood pressure
OD: Outer diameter
PE´CO_2_: End-tidal partial pressure of carbon dioxide
SC: Subcutaneously
SpO_2_: Peripheral oxygen saturation
VL: Video laryngoscope
